# ﻿A new species of *Raorchestes* (Anura, Rhacophoridae) from Yunnan Province, China

**DOI:** 10.3897/zookeys.1192.106013

**Published:** 2024-02-22

**Authors:** Lingyun Du, Yuhan Xu, Shuo Liu, Guohua Yu

**Affiliations:** 1 Key Laboratory of Ecology of Rare and Endangered Species and Environmental Protection, Guangxi Normal University, Ministry of Education, Guilin 541004, China; 2 Guangxi Key Laboratory of Rare and Endangered Animal Ecology, College of Life Science, Guangxi Normal University, Guilin 541004, China; 3 Kunming Natural History Museum of Zoology, Kunming Institute of Zoology, Chinese Academy of Sciences, Kunming 650223, China

**Keywords:** Indochina, “*Raorchestesgryllus*”, *Raorchesteshekouensis* sp. nov., species diversity, taxonomy

## Abstract

A new bush frog species is described from Yunnan, China, based on phylogenetic analyses, species delimitation analyses, and morphological comparisons. *Raorchesteshekouensis***sp. nov.** is distinguished from all other congeners by a combination of 11 morphological characters. The new species brings the current number of *Raorchestes* species in China to ten, nine of which are distributed in Yunnan. Molecular analyses supported an unnamed lineage previously recorded as “*Raorchestesgryllus*” in northern Vietnam. Further studies including additional samples are necessary to clarify the species diversity and boundaries of *Raorchestes* in China and Indochina.

## ﻿Introduction

The genus *Raorchestes* Biju, Shouche, Dubois, Dutta & Bossuyt, 2010, which currently contains 76 species (Frost 2023), is one of the most speciose genera within the family Rhacophoridae. Members of *Raorchestes* are characterized by a small body size (15–45 mm), lack of vomerine teeth, transparent/translucent vocal sac when calling, and direct development (Biju et al. 2010; Vijayakumar et al. 2014). *Raorchestes* is widely distributed in South and Southeast Asia, from India to Nepal, Myanmar, Thailand, and Laos to southwestern China, Vietnam, Cambodia, and West Malaysia (Frost 2023).

Most *Raorchestes* species were initially assigned to the genus *Philautus* Gistel, 1848 (Bossuyt and Dubois 2001); however, Yu et al. (2009) and Li et al. (2009) revealed that frogs traditionally classified in *Philautus* consisted of two groups rather than being a monophylum, and Li et al. (2009) proposed the name *Pseudophilautus* Laurent, 1943 for the group primarily distributed on the Indian subcontinent, which itself consists of two reciprocally monophyletic groups, i.e., a radiation with notably large diversity in the Western Ghats of India and a radiation with large diversity in Sri Lanka. Biju et al. (2010) later erected the genus *Raorchestes* for the clade with substantial diversity in the Western Ghats to distinguish it from *Pseudophilautus* sensu stricto, a clade of 80 species largely restricted to Sri Lanka (Biju et al. 2010; Meegaskumbura et al. 2019). Based on phylogenetic analysis, Li et al. (2013) suggested that *Raorchestes* and *Pseudophilautus* formed a sister group of *Kurixalus* Ye, Fei & Dubois, 1999; however, more recent studies based on wider genus-level sampling suggested *Raorchestes* is sister to *Pseudophilautus* (Vijayakumar et al. 2014; Chan et al. 2018; Garg et al. 2021) or the clade composed of *Raorchestes* and *Pseudophilautus* is sister to *Mercurana* Abraham, Pyron, Ansil, Zachariah & Zachariah, 2013 (Meegaskumbura et al. 2019).

As one of the most diverse groups in the Rhacophoridae family, *Raorchestes* frogs form a distinct radiation with more than 80% of the known species distributed in South Asia, especially in India. As such, most research attention has been paid to the taxonomy and evolution of Indian *Raorchestes*. For examples, Vijayakumar et al. (2014) reported on *Raorchestes* relationships within the Western Ghats, naming nine species and recognizing 15 clades within the Western Ghats complex; Vijayakumar et al. (2016) revealed that geological processes, Quaternary glaciations, and ecological gradients drove diversification of *Raorchestes* frogs in the Western Ghats; and Garg et al. (2021) named five species in the Western Ghats and delimited *Raorchestes* into 16 species groups.

The diversity of *Raorchestes* in southwestern China, Indochina, the Himalayas, and northeastern India is markedly lower than that in the Western Ghats. To date, only 16 species are known from these areas, including *R.andersoni* (Ahl, 1927), *R.annandalii* (Boulenger, 1906), *R.cangyuanensis* Wu, Suwannapoom, Xu, Murphy & Che, 2019, *R.dulongensis* Wu, Liu, Gao, Wang, Li, Zhou, Yuan & Che, 2021, *R.gryllus* (Smith, 1924), *R.hillisi* Jiang, Ren, Guo, Wang & Li, 2020, *R.huanglianshan* Jiang, Wang, Ren & Li, 2020, *R.longchuanensis* (Yang & Li, 1978), *R.malipoensis* Huang, Liu, Du, Bernstein, Liu, Yang, Yu & Wu, 2023, *R.manipurensis* (Mathew & Sen, 2009), *R.menglaensis* (Kou, 1990), *R.parvulus* (Boulenger, 1893), *R.rezakhani* Al-Razi, Maria & Muzaffar, 2020, *R.sahai* (Sarkar & Ray, 2006), *R.shillongensis* (Pillai & Chanda, 1973), and *R.yadongensis* Zhang, Shu, Liu, Dong & Guo, 2022 (Frost 2023). Of these 16 species, nine are known in China (i.e., *R.andersoni*, *R.cangyuanensis*, *R.dulongensis*, *R.hillisi*, *R.huanglianshan*, *R.longchuanensis*, *R.malipoensis*, *R.menglaensis*, and *R.yadongensis*), all from the border areas of Yunnan, except for *R.yadongensis*, which is only known from southern Tibet (Zhang et al. 2022). Moreover, the distribution of the *R.andersoni* is also recorded in southern Medog, Tibet (e.g., Chen et al. 2020; Fei et al. 2012), *R.andersoni* was originally described “on level marshy flats on the banks of the Nampoung [= Nanben River] in the centre of the Kakhyen Hills”, Yingjiang County, Yunnan, China by Anderson (1878), and it was once recognized as *Thelodermaandersoni* by Li et al. (2009). However, Hou et al. (2017) suggested that it possibly belonged to *Raorchestes* on the basis of morphological similarities to *Philautuslongchuanensis*, subsequently Chen et al. (2020) transferred *T.andersoni* (Ahl, 1927) to the genus *Raorchestes* based on the molecular evidence. *Raorchestesparvulus* was originally described from Karin Bia-po in Myanmar by Boulenger (1893) and previously recorded from China by Yu et al. (2019) based on specimens from Menglun, Yunnan. However, Jiang et al. (2020) considered that the record of *R.parvulus* from Yunnan was misidentified and revised it to *R.menglaensis*.

Six *Raorchestes* species are known from Southeast Asia, i.e., *R.parvulus* (Boulenger, 1893), *R.gryllus*, *R.menglaensis*, *R.huanglianshan*, *R.longchuanensis*, and *R.malipoensis* (Frost 2023). However, the taxonomic status of *R.gryllus* is problematic. This species was originally described from Langbian Plateau in Lam Dong Province, southern Vietnam, and has been widely reported in Vietnam (Lam Dong, Dak Lak, Gia Lai, and Kon Tum, Lao Cai, Cao Bang, Vinh Phu, and Bac Thai) and Laos (Sepian, Boloven Highlands, Champasak Province) (Bourret 1937, 1939, 1942; Orlov et al. 2002, 2012; Teynié et al. 2004; Nguyen et al. 2009). Biju et al. (2010) confirmed the affiliation of *R.gryllus* with the genus *Raorchestes* based on molecular data from Li et al. (2009). However, those specimens used in Li et al. (2009) were collected from Pac Ban, Tuyen Quang, northern Vietnam, and Orlov et al. (2012) considered records of *R.gryllus* in this region to be highly improbable. Furthermore, the species contains a series of tubercles along the outer side of the forearm and foot, and a dermal projection on the snout (Smith 1924), very similar to members of *Kurixalus*, and differing in egg capsule appearance from other *Raorchestes* species, with thick and semi-transparent eggs in *R.gryllus* compared to transparent eggs in other *Raorchestes* species (Orlov et al. 2012).

Recently, Poyarkov et al. (2021) suggested the transfer of *R.gryllus* to *Kurixalus* based on unpublished molecular data of specimens from the type locality and unpublished morphological data from type material, implying that the so-called “*R.gryllus*” specimens from northern Vietnam used in previous phylogenetic analyses (e.g., Li et al. 2009, 2013; Nguyen et al. 2014; Wu et al. 2019) are not actually true *K.gryllus*, but represent an unnamed species. Moreover, Huang et al. (2023) considered that the specimen of *R.* UI ROM30288 from Pac Ban, Tuyen Quang, northern Vietnam was misidentified and revised it to *R.malipoensis*. This suggests that other records of the species from Vietnam and Laos need further examination.

Yunnan Province harbors the highest amphibian species diversity in China (AmphibiaChina, 2022), with many new species described in recent years (e.g., Gan et al. 2020; Liu et al. 2021; Wang et al. 2022). During recent field surveys in Hekou, Yunnan, China, we collected eight specimens of *Raorchestes*. Morphological comparison and phylogenetic analysis indicated that these specimens could be distinguished from all other members of the genus *Raorchestes*, except for the *R.* UI ROM 38828 from northern Vietnam in molecular analysis, indicating that the eight specimens from Hekou and the ROM38828 specimen from northern Vietnam represent a new species.

## ﻿Materials and methods

### ﻿Sampling

**Figure 1.**
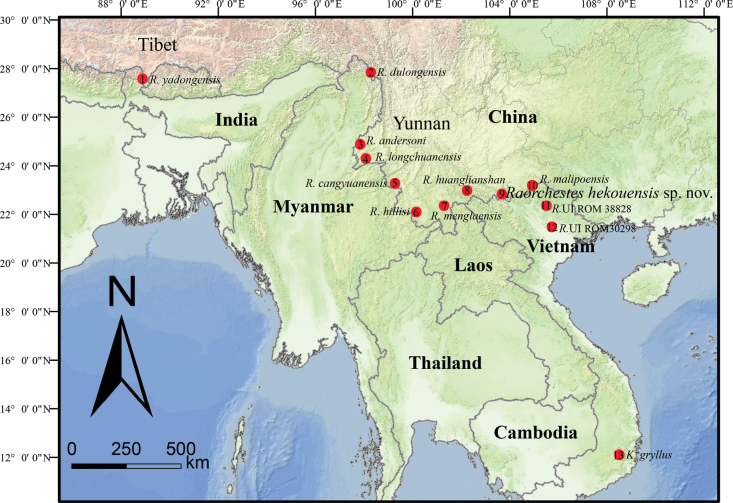
Map showing type localities of *Raorchestes* species originally described from China (1–10), type locality of *K.gryllus* in Vietnam (13), and collection sites of *R.* UI used in this study (11, 12). *Raorchesteshekouensis* sp. nov. is known from the type locality (9) and Pac Ban, Tuyen Quang, Vietnam (11).

Field surveys were conducted in March 2019 and April 2023 at Liangzi village, Hekou, Yunnan, China (Fig. 1). Specimens were euthanized, fixed, and preserved in 75% ethanol. Liver tissues were taken and preserved in 99% ethanol. Voucher specimens and tissue samples were deposited at Guangxi Normal University (**GXNU**), China.

### ﻿Morphology and morphometrics

All measurements were made with slide calipers to the nearest 0.1 mm. Morphological characters and measurements followed Du et al. (2020) and included: snout-vent length (**SVL**); head length (**HL**); head width (**HW**); snout length (**SL**); internarial distance (**INS**); interorbital distance (**IOS**); maximum transverse distance of upper eyelid (**UEW**); eye diameter (**ED**); tympanum diameter (**TD**); eye-nostril distance (**EN**); length of lower arm and hand (**LAHL**); tibia length (**TIL**); length of foot and tarsus (**TFL**); foot length (**FL**). Morphological measurements of the specimens are given in Table 1. Males and females were identified based on the presence of an external single subgular vocal sac or sac slit opening. Comparative data on the morphology of other *Raorchestes* species were obtained from previous publications (Boulenger 1893, 1906; Smith 1924; Pillai and Chanda 1973; Yang and Li 1978; Kou 1990; Bossuyt and Dubois 2001; Sarkar and Ray 2006; Fei et al. 2009, 2012; Mathew and Sen 2009; Orlov et al. 2012; Wu et al. 2019, 2021; Al-Razi et al. 2020; Che et al. 2020; Jiang et al. 2020; Zhang et al. 2022; Huang et al. 2023).

**Table 1.** Measurements (in mm) of *Raorchesteshekouensis* sp. nov. specimens from Liangzi, Hekou, Yunnan. Holotype is marked with an asterisk (*).

Catalog No.	Adults					Sub-adults		
	GXNU YU000159*	GXNU YU000536	GXNU YU000537	GXNU YU000538	GXNU YU000160	GXNU YU000153	GXNU YU000154	GXNU YU000156
Sex	Male	Male	Male	Male	Female	Male	Female	Female
SVL	17.5	17.8	16.7	16.1	21.1	14.5	12.5	12.9
HL	6.1	5.8	5.7	5.6	7.2	5.1	4.1	4.5
HW	6.9	7.1	6.1	6.1	7.6	5.3	4.9	4.5
SL	2.4	1.7	1.6	1.9	2.8	1.7	1.3	1.6
INS	2.2	2.4	2.1	2.3	2.6	2.0	1.6	1.8
IOS	2.4	2.8	2.3	2.1	2.7	2.0	1.7	1.8
UEW	1.9	1.7	1.9	1.5	2.1	1.2	1.1	1.7
ED	2.5	3.1	2.7	2.4	3.0	2.3	2.0	2.0
TD	1.3	1.1	1.3	1.2	1.4	0.7	0.5	0.8
DNE	1.5	1.2	1.3	1.3	1.8	1.2	1.0	1.0
LAHL	8.5	7.4	7.9	7.1	10.1	6.9	5.8	6.0
TIL	9.2	8.8	8.4	7.8	10.6	7.7	6.1	6.1
TFL	11.4	11.0	10.4	8.9	13.6	8.8	7.8	7.5
FL	6.6	6.6	5.7	5.4	8.1	5.1	4.1	4.1

### ﻿DNA sequencing

We extracted genomic DNA from liver tissues stored in 99% ethanol following standard protocols (Vences et al. 2012). We amplified and sequenced the mitochondrial 16S ribosomal RNA (16S) genes using the primer pair L2188 (Matsui et al. 2006) and 16H1 (Hedges, 1994). Polymerase chain reaction (PCR) amplifications were performed in a 50-μL reaction volume, with an initial denaturing step at 95 °C for 4 min, 35 cycles of denaturing at 94 °C for 1 min, annealing at 51 °C for 1 min, and extension at 72 °C for 1 min, with a final extension step of 72 °C for 10 min. Sequencing was conducted using the corresponding PCR primers. All new sequences were deposited in GenBank under accession numbers ON986419–ON986422, OQ029526, OQ859106 and OQ859107 (Table 2).

**Table 2.** Information on voucher numbers, localities, and GenBank accession numbers for all specimens used in this study.

Species	Locality	Voucher No.	16S	Reference
*Raorchesteshekouensis* sp. nov.	Hekou, Yunnan, China	GXNU YU000153	ON986419	This study
*Raorchesteshekouensis* sp. nov.	Hekou, Yunnan, China	GXNU YU000154	OQ029526	This study
*Raorchesteshekouensis* sp. nov.	Pac Ban, Tuyen Quang, Vietnam	ROM 38828	KC465838	Li et al. (2013)
*Raorchesteshekouensis* sp. nov.	Hekou, Yunnan, China	GXNU YU000156	ON986420	This study
*Raorchesteshekouensis* sp. nov.	Hekou, Yunnan, China	GXNU YU000159	ON986421	This study
*Raorchesteshekouensis* sp. nov.	Hekou, Yunnan, China	GXNU YU000160	ON986422	This study
*Raorchesteshekouensis* sp. nov.	Hekou, Yunnan, China	GXNU YU000536	OQ859106	This study
*Raorchesteshekouensis* sp. nov.	Hekou, Yunnan, China	GXNU YU000537	OQ859107	This study
*Raorchestesandersoni*	Medog, Tibet, China	KIZYPX16167	MW023609	Chen et al. (2020)
*Raorchestesandersoni*	Medog, Tibet, China	KIZ014104	MW023610	Chen et al. (2020)
*Raorchestesannandalii*	Nepal	CDZMTU419	MT983169	Khatiwada et al. (2021)
*Raorchestesagasthyaensis*	Western Ghats, India	CESF492	JX092723	Vijayakumar et al. (2014)
*Raorchestesarcheos*	Agasthyamalai Massif, Western Ghats, India	CESF1190	JX092675	Vijayakumar et al. (2014)
*Raorchestescangyuanensis*	Cangyuan, Yunnan, China	KIZ 015855	MN475866	Wu et al. (2019)
*Raorchestescangyuanensis*	Cangyuan, Yunnan, China	KIZ 015856	MN475867	Wu et al. (2019)
*Raorchestescrustai*	Elivalmalai Massif, Western Ghats, India	CESF1199	JX092677	Vijayakumar et al. (2014)
*Raorchesteschromasynchysi*	Western Ghats, India	CESF1127	JX092667	Vijayakumar et al. (2014)
*Raorchestesdulongensis*	Qinlangdang, Yunnan, China	KIZ 035082	MW537814	Wu et al. (2019)
*Raorchestesdulongensis*	Qinlangdang, Yunnan, China	KIZ0 35125	MW537815	Wu et al. (2019)
*Raorchestesghatei*	Western Ghats, India	CESF1262	JX092687	Vijayakumar et al. (2014)
*Raorchestes* UI	Tam Dao, Vinh Phuc, Vietnam	ROM 30298	MN475869	Wu et al. (2019)
*Raorchesteshillisi*	Xiding, Yunnan, China	CIB116329	MT488412	Jiang et al. (2020)
*Raorchesteshillisi*	Xiding, Yunnan, China	CIB116330	MT488413	Jiang et al. (2020)
*Raorchesteshuanglianshan*	Lvchun, Yunnan, China	CIB116353	MT488415	Jiang et al. (2020)
*Raorchesteshuanglianshan*	Lvchun, Yunnan, China	CIB116354	MT488417	Jiang et al. (2020)
*Raorchestesleucolatus*	Elivalmalai Massif, Western Ghats, India	CESF1147	JX092669	Vijayakumar et al. (2014)
*Raorchesteslongchuanensis*	Gongdong, Yunnan, China	KIZ 048468	MN475870	Wu et al. (2019)
*Raorchesteslongchuanensis*	Gongdong, Yunnan, China	KIZ048492	MN475871	Wu et al. (2019)
*Raorchestesmalipoensis*	Pac Ban, Tuyen Quan, Vietnam	ROM30288	GQ285674	Li et al. (2009)
*Raorchestesmalipoensis*	Malipo, Yunnan, China	GXNU 000339	ON128245	Huang et al. (2023)
*Raorchestesmenglaensis*	Zhushihe, Yunnan, China	CIB116338	MT488403	Jiang et al. (2020)
*Raorchestesmenglaensis*	Zhushihe, Yunnan, China	CIB116340	MT488404	Jiang et al. (2020)
*Raorchestesparvulus*	Pulau Langkawi, Malaysia	LSUHC 7596	MH590202	Chan et al. (2018)
*Raorchestesparvulus*	Gunung Stong, Malaysia	LSUHC 11118	MH590201	Chan et al. (2018)
*Raorchestesrezakhani*	Maulovibazar, Bangladesh	JnUZool-A0319	MN072374	Al-Razi et al. (2020)
*Raorchestesshillongensis*	Malki forest, Shilong, Meghalaya, India	R2	MG980283	Unpublished
*Raorchestes* sp. 1	India	CESF420	JX092712	Vijayakumar et al. (2014)
*Raorchestestuberohumerus*	Western Ghats, India	0073PhiTub	EU450004	Biju and Bossuyt, (2009)
*Raorchestesuthamani*	Western Ghats, India	CESF483	JX092722	Vijayakumar et al. (2014)
*Raorchestesyadongensis*	Yadong, Xizang, China	YBU 21222	OP345440	Zhang et al. (2022)
*Raorchestesyadongensis*	Yadong, Xizang, China	YBU 21223	OP345441	Zhang et al. (2022)
*Pseudophilautuskani*	Western Ghats, India	CESF497	JX092724	Vijayakumar et al. (2014)
*Pseudophilautusamboli*	Western Ghats, India	BNHS4399	EU450025	Biju and Bossuyt (2009)

### ﻿Phylogenetic analysis and species delimitation

To examine the phylogenetic position of the specimens collected from Hekou, Yunnan, China, we reconstructed phylogenetic trees of the genus *Raorchestes* based on sequences of the 16S rRNA (16S) genes. Furthermore, 35 homologous sequences of other *Raorchestes* species were obtained from GenBank (Table 2). *Pseudophilautuskani* (Biju & Bossuyt, 2009) and *Pseudophilautusamboli* (Biju & Bossuyt, 2009) were selected as outgroups based on Wu et al. (2021). All sequences were aligned in MEGA v. 7.0 (Kumar et al. 2016) using the ClustalW tool and both ends of the sequence were trimmed to minimize missing characters.

Phylogenetic relationships were inferred based on maximum likelihood (ML) and Bayesian inference (BI) analyses. BI analysis was conducted in MrBayes v. 3.2.6 (Ronquist et al. 2012). The best-fitting model (GTR + I + G) was chosen using the Akaike Information Criterion (AIC) in JModelTest v. 2.1.10 (Darriba et al. 2012). Four Monte Carlo Markov chains were started from a random tree. The chains were run for three million generations and sampled every 100 generations, with the first 25% of sampled trees discarded as burn-in. The remaining trees were used to create a consensus tree and to estimate Bayesian posterior probabilities (BPP). ML analysis was performed using RAxML v. 8.2.10 (Stamatakis 2014) under the GTR + I + G model. Tree searches were performed 100 times with 1000 bootstrap (BS) replicates to assess node support. Nodes with BPP ≥ 0.95 and BS ≥ 70 were considered well supported. Additionally, uncorrected pairwise genetic distances (*p*-distances) between species in 16S rRNA sequences were calculated using MEGA v. 7.0. (Kumar et al. 2016).

We used two approaches, i.e., the Bayesian Poisson Tree Processes (bPTP; Zhang et al. 2013) and Assemble Species by Automatic Partitioning (ASAP; Puillandre et al. 2021), to delimit species boundaries. The bPTP method was run on the bPTP server (http://species.h-its.org/) using the tree generated by Bayesian phylogenetic analysis and default parameters. For the ASAP method, the simple distance (*p*-distance) model was used and the partitioning with the lowest ASAP score was selected as the best, as per Puillandre et al. (2021).

## ﻿Results

### ﻿Phylogenetic analysis and genetic divergence

**Figure 2.**
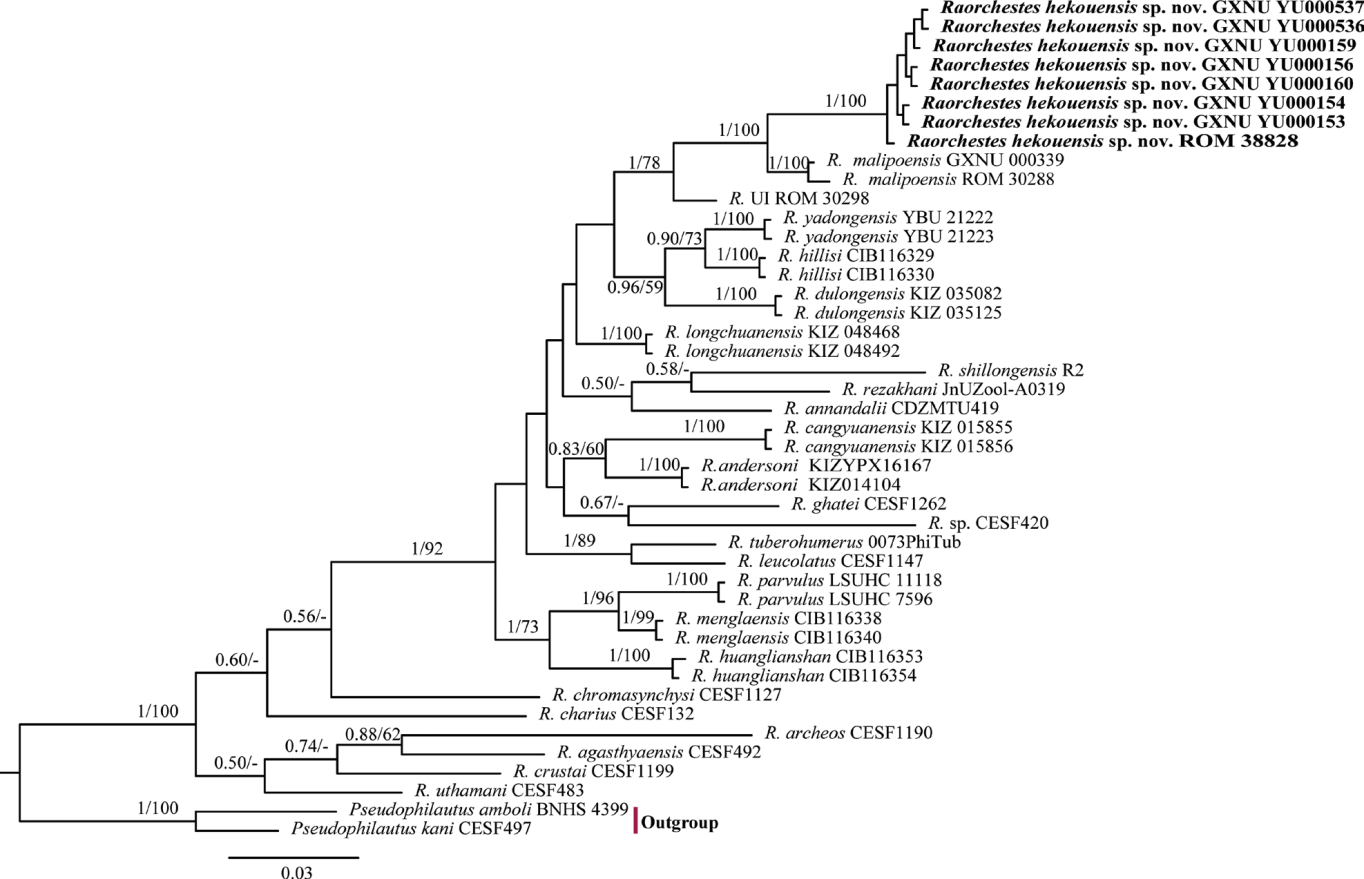
Bayesian phylogram of *Raorchestesparvulus* group estimated from 16S rRNA showing placement of *Raorchesteshekouensis* sp. nov. Nodal support values are shown above branches as Bayesian posterior probability (BPP) / ML bootstrap support (BS), and the symbol “-” indicates value below 50.

**Figure 3.**
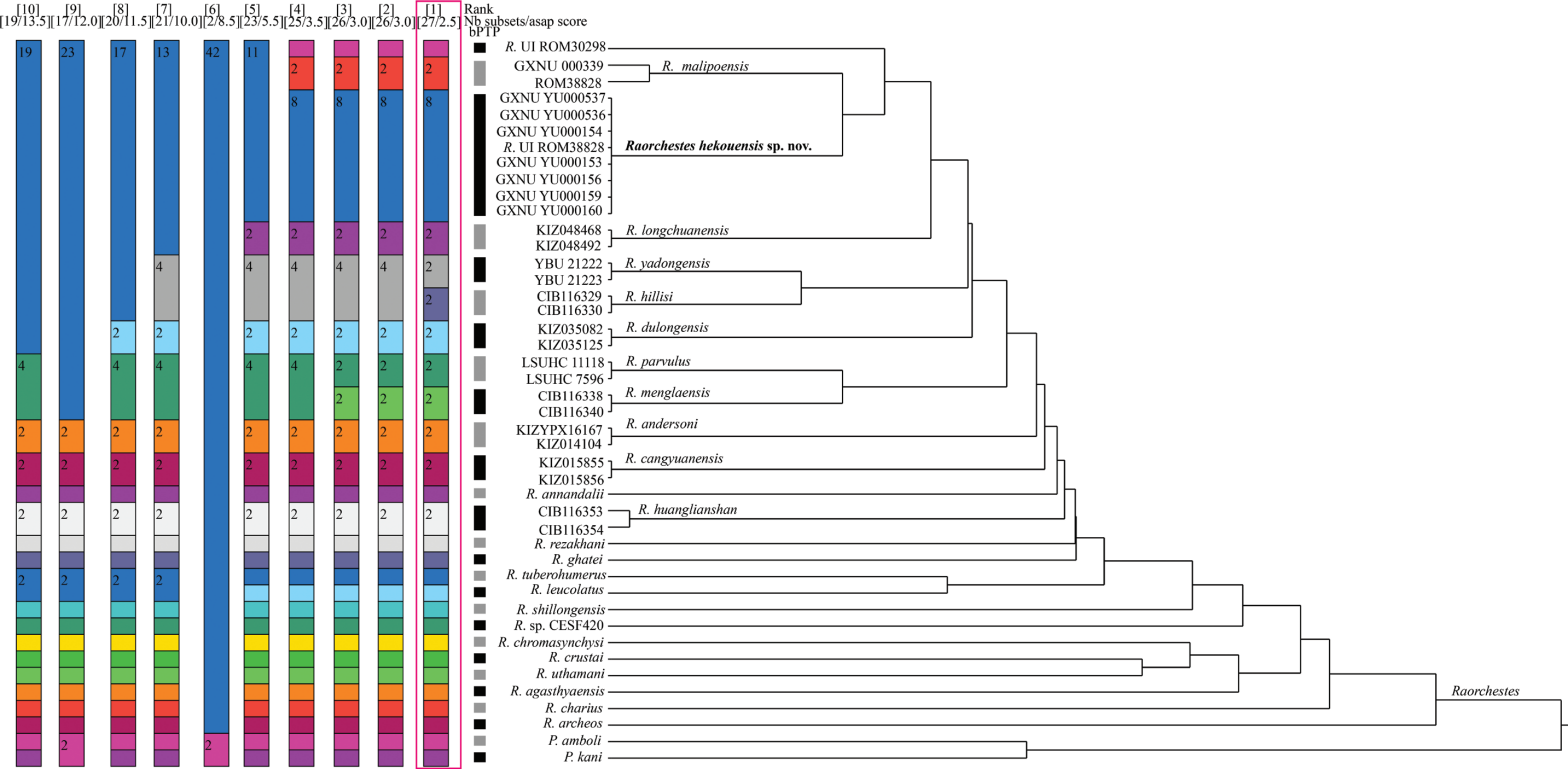
ASAP species delimitation within *Raorchestes* based on 16S sequences. ASAP analysis generated 10 partitions and ranked them using the lowest ASAP score as the best option, and the best partition is highlighted in red. Black and gray vertical bars indicate results of bPTP species delimitation.

The obtained sequence alignment was 552 bp long and included 211 variable sites and 152 parsimony informative sites. Phylogenetic analysis (Fig. 2) revealed that the specimens from Hekou, Yunnan, China, *R.malipoensis* and *R.* UI from northern Vietnam formed a monophyletic group, which itself contained three distinct branches, one consisting of the specimens from Hekou and a specimen of *R.* UI from Pac Ban, Tuyen Quang, Vietnam (ROM 38828) with strong support (BPP = 100, BS = 100) and short internal branch lengths, one consisting only of *R.* UI from Tam Dao, Vinh Phuc, Vietnam (ROM 30298), and one consisting of the recently named bush frog species *R.malipoensis*, which included a specimen previously mistaken of “*R.gryllus*” from Pac Ban, Tuyen Quang, Vietnam (ROM 30288). The clade containing specimens from Hekou was recovered as the sister to *R.malipoensis* with strong support. The bPTP analysis delimited the three lineages into three candidate species (Fig. 3). The ASAP analysis identified 10 partitions (Fig. 3) and the best partition (score = 2.5) also grouped the three lineages into three candidate species. The 16S*p*-distances between the clade consisting of Hekou specimens and the other *Raorchestes* lineages included in this study ranged from 2.5% (*R.malipoensis*) to 12.9% (*R.archeos*), greater than the divergence between *R.hillisi* and *R.yadongensis* (2.0%; Table 3).

**Table 3.** Uncorrected p-distance (%) in 16S rRNA sequences of *Raorchestes* species used in this study.

ID	Species	1	2	3	4	5	6	7	8	9	10	11	12	13	14	15	16	17	18	19	20	21	22	23	24	25
1	*R.hekouensis* sp. nov.																									
2	*R.malipoensis*	2.5																								
3	*R.* UI ROM30298	3.7	2.9																							
4	*R.longchuanensis*	4.1	3.5	3.2																						
5	*R.rezakhani*	5.3	5.0	4.6	4.8																					
6	*R.andersoni*	5.4	4.9	4.9	4.4	5.2																				
7	*R.tuberohumerus*	6.0	6.2	5.4	5.3	6.6	6.5																			
8	*R.menglaensis*	6.0	5.5	4.7	4.4	6.7	5.9	5.5																		
9	*R.annandalii*	6.1	5.7	5.3	4.6	5.1	4.4	7.0	6.4																	
10	*R.hillisi*	6.1	5.2	3.5	4.5	5.2	5.3	5.6	5.5	5.5																
11	*R.parvulus*	6.1	6.8	6.9	5.2	8.2	6.7	7.3	2.5	6.5	7.3															
12	*R.dulongensis*	6.2	5.7	3.7	3.9	5.6	4.8	6.8	5.8	5.9	3.7	7.5														
13	*R.leucolatus*	6.6	6.1	5.9	5.1	7.3	6.2	3.3	5.3	6.4	6.5	5.9	7.3													
14	*R.yadongensis*	6.7	5.1	3.9	4.4	6.0	4.9	5.5	5.5	5.3	2.0	6.7	3.7	5.7												
15	*R.ghatei*	7.1	6.7	5.2	5.4	5.7	5.0	5.7	6.5	5.9	5.7	7.7	5.8	4.9	5.2											
16	*R.cangyuanensis*	7.6	6.6	5.8	5.8	6.5	4.4	7.3	5.8	6.0	6.2	7.6	6.4	6.9	5.4	6.5										
17	*R.huanglianshan*	7.6	6.8	6.0	5.5	6.3	6.3	7.1	4.7	6.7	5.8	5.5	6.1	7.0	5.6	6.9	6.4									
18	*R.* sp 1	8.5	8.0	7.2	6.3	7.0	8.1	8.1	9.0	7.6	8.1	10.6	7.8	8.0	8.0	7.7	9.7	9.9								
19	*R.uthamani*	8.7	7.9	8.6	8.2	9.0	8.3	8.9	8.6	9.2	8.5	8.6	8.8	6.8	8.3	9.1	9.9	9.0	11.5							
20	*R.shillongensis*	8.9	8.0	7.3	6.8	5.8	7.3	8.5	8.0	7.0	7.9	9.2	7.7	7.8	7.5	8.4	9.8	8.2	7.4	10.6						
21	*R.charius*	9.2	9.3	9.1	8.4	8.6	8.9	7.8	8.1	10.1	9.2	8.9	9.7	8.0	8.9	8.5	9.8	7.7	10.9	7.9	10.4					
22	*R.agasthyaensis*	9.7	9.3	9.3	9.4	9.6	9.3	9.1	9.0	11.0	8.5	9.4	9.4	7.8	9.0	10.4	10.9	9.9	12.5	6.8	11.9	9.2				
23	*R.chromasynchysi*	9.8	9.1	9.1	7.4	8.8	7.8	9.4	8.7	9.7	8.5	9.1	8.0	7.8	7.4	7.2	8.8	8.6	10.3	5.7	9.5	7.1	7.3			
24	*R.crustai*	12.6	11.4	10.3	11.6	10.6	10.7	10.4	10.7	12.8	9.6	11.4	10.3	9.4	9.1	10.2	11.8	10.4	14.7	5.3	13.0	7.9	6.2	9.5		
25	*R.archeos*	12.9	11.2	12.5	12.2	12.1	12.4	12.6	11.3	12.2	11.1	11.8	12.6	11.8	10.1	13.5	12.8	11.7	14.7	9.1	15.8	10.1	8.2	10.1	9.2	

### ﻿Taxonomic account

#### ﻿ Raorchestes hekouensis sp. nov.

Taxon classificationAnimaliaAnuraRhacophoridae

3E4731DD-A46D-5E36-A4A1-1751669D45AA

https://zoobank.org/4175879C-5620-49B0-B016-8489657C6069

**Figure 4.**
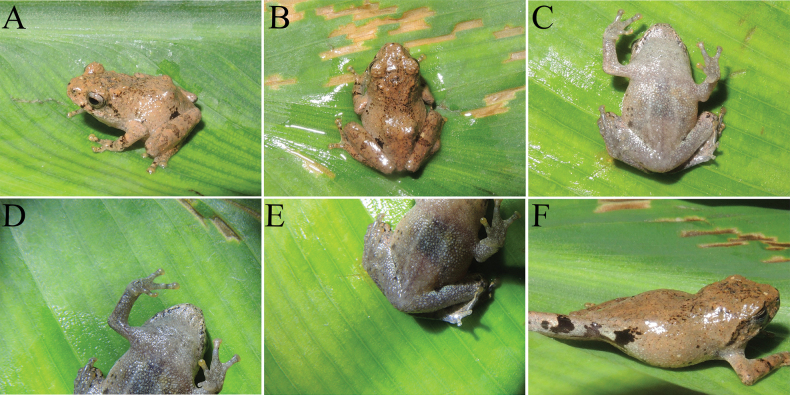
Photographs of holotype of *Raorchesteshekouensis* sp. nov. (GXNU YU000159) in life. Lateral view (**A**), dorsal view (**B**), ventral view (**C**), fingers (**D**), toes (**E**), crotch (**F**).

**Figure 5.**
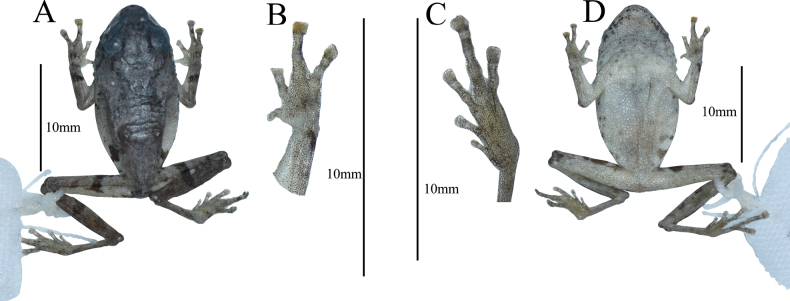
Photographs of *Raorchesteshekouensis* sp. nov. holotype (GXNU YU000159) in preservative, dorsal view (**A**), ventral view of hand (**B**), ventral view of foot (**C**), ventral view (**D**).

**Figure 6.**
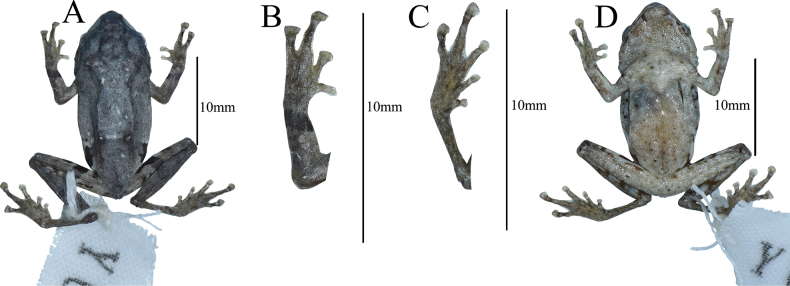
Photographs of *Raorchesteshekouensis* sp. nov. paratype (GXNU YU000160) in preservative, dorsal view (**A**), dorsal view of hand (**B**), ventral view of foot (**C**), ventral view (**D**).

**Figure 7.**
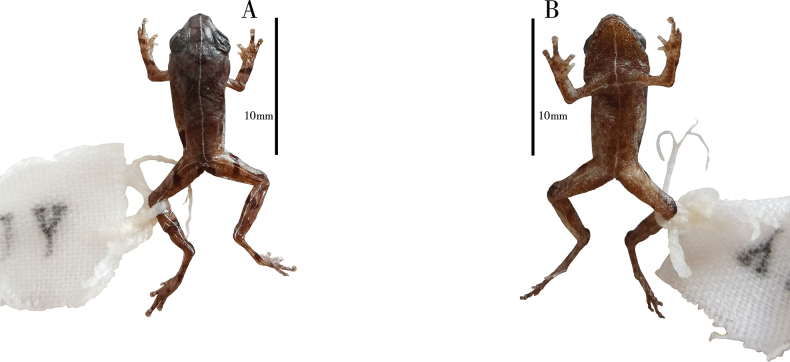
Photographs of *Raorchesteshekouensis* sp. nov. paratype (GXNU YU000156) in preservative, dorsal view (**A**), and ventral view (**B**).

**Figure 8.**
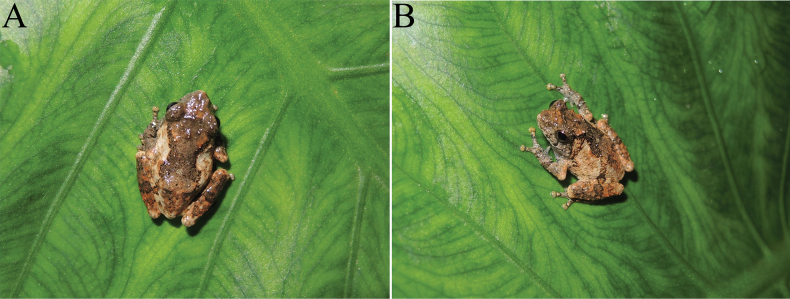
Photographs of *Raorchesteshekouensis* sp. nov. paratype (GXNU YU000537) in life, dorsal view (**A**), and lateral view (**B**).

Table 1 , Figs 4 , 5 , 6 , 7 , 8

##### Chresonymy.

*Raorchestesgryllus* (Li et al. 2013).

##### Type material.

***Holotype*.** GXNU YU000159, adult male, collected on 25 March 2019 by Shuo Liu from Liangzi, Hekou, Yunnan, China (22°49'N, 103°44′E, 1200 m a.s.l.; Fig. 1).

***Paratypes*.** Adult female (GXNU YU000160), three sub-adults (GXNU YU000153, GXNU YU000154, and GXNU YU000156) with the same collection information as the holotype, and three adult males (GXNU YU000536, GXNU YU000537 and GXNU YU000538) collected at the same locality as the holotype on 4 April 2023 by Lingyun Du and Shuo Liu.

##### Etymology.

The specific epithet *hekouensis* is named after the type locality, Hekou County, Yunnan, China. We suggest “Hekou bush frog” as its English common name, and “Hé Kǒu Guàn Shù Wā (河口灌树蛙)” as its Chinese common name.

##### Diagnosis.

*Raorchesteshekouensis* sp. nov. is distinguished from all other relevant congeners by a combination of the following characters: (1) small body size (male SLV 16.1–17.5 mm, *n* = 4; female 21.1 mm, *n* = 1); (2) tympanum distinct; (3) tips of all fingers and toes expanded into discs with circummarginal grooves; (4) rudimentary webbing on toes; (5) all fingers and toes with lateral dermal fringes; (6) inner metacarpal tubercle present and outer metacarpal tubercle indistinct; (7) heels meeting when limbs held at right angles to body; (8) discs of fingers and toes yellow; (9) male with external single subgular vocal sac; (10) distinct X-shaped dark brown marking on back; (11) inner metatarsal tubercle oval, outer metatarsal tubercle absent.

##### Description of holotype.

GXNU YU000159, adult male, body size small (SVL 17.5 mm); head wider than long (HW = 6.9 mm, HL = 6.1 mm); snout rounded in profile, projecting beyond lower jaw, snout length almost equal to diameter of eye (SL = 2.4 mm; ED = 2.5 mm); canthus rostralis rounded, loreal region slightly concave; internarial distance slightly less than interorbital distance, and wider than maximum width of upper eyelid (INS = 2.2 mm; IOS = 2.4 mm; UEW = 1.9 mm); tympanum distinct (TD = 1.3 mm); tongue pyriform, with deep notch at posterior tip; vomerine teeth absent; temporal fold distinct; dorsolateral fold absent. Length of forelimb and hand slightly shorter than half of snout-vent length (LAHL = 8.5 mm, SVL = 17.5); relative fingers lengths: I < II < IV < III; tips of all four fingers expanded into discs with circummarginal grooves; lateral dermal fringes on all fingers; subarticular tubercles distinct, rounded; supernumerary tubercles absent; no webbing between fingers; inner metacarpal tubercle present, outer metacarpal tubercle indistinct; nuptial pads present on first and second fingers in male. Hindlimbs relatively slender, thigh length (TIL = 9.2) shorter than tibia length (TL = 11.4), but greater than foot length (FL = 6.6); tibiotarsal articulation reaching anterior of eye when hindlimb stretched alongside body; heels meeting when limbs held at right angles to body; relative toe lengths: I < II < III < V < IV; tips of toes with well-developed discs with circummarginal grooves; all toes with lateral dermal fringes; subarticular tubercles distinct, rounded; supernumerary tubercles absent; rudimentary webbing between toes; inner metatarsal tubercle rounded, outer metatarsal tubercle absent. Dorsal surfaces rough, dorsum, dorsal surface of limbs, snout, between eyes, and upper eyelid shagreened with numerous tubercles; flank of body, dorsal part of forelimbs, thighs, and tibia relatively smooth, scattered with sparse granules; throat, chest, and ventral surfaces of forelimbs smooth; abdomen, underside of thigh, and around vent with granules; dorsolateral folds absent; dorsal, dorsal surface of limbs and around vent with several beige patches.

##### Coloration of holotype in life.

Dorsal surface yellowish brown, with distinct dark brown X-shaped marking on back; blackish line between eyes; tea-brown spots on both sides of lower jaw; dorsal side of limbs with several brown bands; flank near crotch with distinct black region between two creamy white patches, thighs with similar black patch near groin, next to another creamy white patch; ventral surface of throat, chest, ventral side of limbs, and belly opaque creamy white with small black spots and white tubercles; finger and toe discs yellow (Fig. 4).

##### Coloration of holotype in preservative.

Dorsal color changed to grayish brown; forelimbs and hindlimbs with black-brown bands; patches or spots blackish brown; abdomen and ventral sides of limbs still milky white with several small black spots (Fig. 5).

##### Male secondary sexual characteristics.

Adult male with nuptial pads on dorsal surface of first and second fingers and external single subgular vocal sac with slit-like opening at posterior of jaw. White lineae masculinae visible on ventral body.

##### Variation.

Specimen GXNU YU000160 significantly has more black spots on the abdomen and near the cloaca (Fig. 6), specimen GXNU YU000156 differs from the other seven type specimens (GXNU YU000159, GXNU YU000160, GXNU YU000153, GXNU YU000154, GXNU YU000536, GXNU YU000537, and GXNU YU000538) by pale yellow mid-dorsal vertebral stripe from snout to vent, pale yellow stripe along hindlimbs crossing at vent region, mid-ventral stripe from snout to vent and stripe along forelimbs crossing at breast region (Fig. 7), and the specimen GXNU YU000537 has distinctly darker ground color on the dorsal side, especially on the head (Fig. 8).

##### Distribution.

Currently known from the type locality, Hekou County, Yunnan Province, China, and Bac Pan, Tuyen Quang, Vietnam.

##### Habitat.

**Figure 9.**
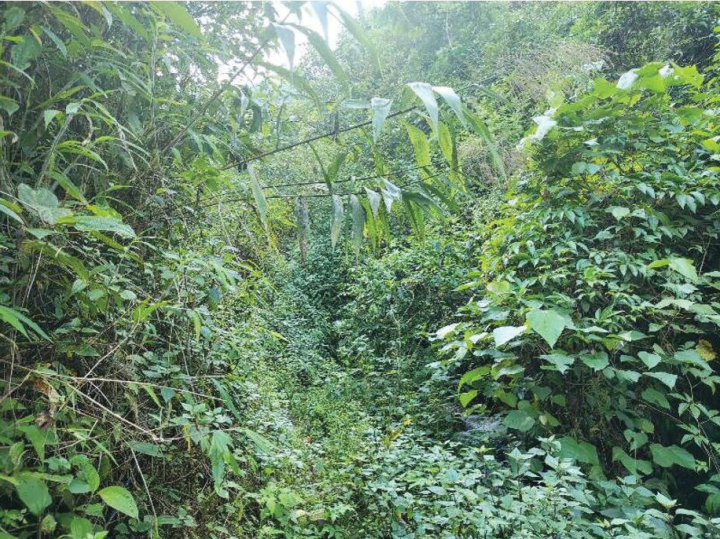
Habitat at type locality of *Raorchesteshekouensis* sp. nov. at Liangzi Village, Hekou, Yunnan, China.

In Yunnan, *Raorchesteshekouensis* sp. nov. was found in shrubs and herbs on the edge of a small stream near the road at an elevation of ca 1200 m a.s.l. (Fig. 9) on the nights of 25 March 2019 and 4 April 2023. There were many herbaceous plants near the stream, such as *Ageratinaadenophora*. No male was heard calling and no eggs were observed during our surveys in late March, but there were males calling during our surveys in April. Therefore, the breeding season for this species starts in April.

##### Remarks.

*Raorchesteshekouensis* sp. nov. is assigned to the genus *Raorchestes* based on its molecular phylogenetic position and the following morphological characters: relatively small body size (SVL 15.0–45.0 mm); absence of vomerine teeth; large transparent/translucent vocal sac. Due to the close phylogenetic relationship and distribution (Figs 1, 2), we compared the new species with 16 recognized congeners distributed in Southeast Asia, southwestern China, the Himalayas, and northeastern India, as mentioned above. *Raorchesteshekouensis* sp. nov. is distinguished from all other 16 congeners by a unique combination of characters. A detailed morphological comparison table of currently known *Raorchestes* species from China is provided (Table 4).

**Table 4.** Morphological comparison among currently known species of *Raorchestes* in China (? = unknown).

Character	*Raorchesteshekouensis* sp. nov.	*R.cangyuanensis*	*R.dulongensis*	*R.menglaensis*	*R.longchuanensis*	*R.huanglianshan*	*R.hillisi*	*R.andersoni*	*R.yadongensis*	*R.malipoensis*
SVL of adult male (mm)	16.1–17.5	16.1–20.0	15.0–19.0	15.0–21.6	17.8–21.2	17.0–19.6	15.9–17.7	24.0	17.8–24.1	14.6–19.3
HDL/HDW	HDL < HDW	HDL < HDW	HDL > HDW	HDL ≈ HDW	HDL ≈ HDW	HDL ≤ HDW	HDL > HDW	HDL < HDW	HDL < HDW	HDL < HDW
Tympanum	Distinct	Indistinct	Distinct	Indistinct	Distinct	Distinct	Distinct	Distinct	Distinct	Distinct
Nuptial pad	Present	Present	Absent	Present	Present	Present	Present	?	Present	Present
Vocal sac	External single subgular vocal sac	External single subgular vocal sac	External single subgular vocal sac	Internal single subgular vocal sac	External single subgular vocal sac	External single subgular vocal sac	External single subgular vocal sac	Internal single subgular vocal sac	External single subgular vocal sac	External single subgular vocal sac
Finger web	Absent	Absent	Absent	Absent	Absent	Absent	Absent	Absent	Rudimentary	Absent
Toe web	Rudimentary	Rudimentary	Rudimentary	Rudimentary or 1/4	1/4 webbing	Rudimentary, except between toe I and toe II	Rudimentary, except between toe I and toe II	Rudimentary or 1/3	Rudimentary	Rudimentary
Outer metatarsal tubercle	Absent	Absent	Absent	Present	Absent	Absent	Absent	Absent	Absent	Absent
Relative toe lengths	I < II < III < V < IV	I < II < V < III < IV	I < II < V < III < IV	I < II < III ≤ V < IV	I < II < III = V < IV	I < II < III < V < IV	I < II < III < V < IV	I < II < III ≤ V < IV	I < II < III < V < IV	I < II < V < III < IV
Reference	This study; Li et al. (2013)	Wu et al. (2019)	Wu et al. (2020)	Kou 1990; Jiang et al. (2020)	Yang and Li 1987; Fei et al. (2009)	Jiang et al. (2020)	Jiang et al. (2020)	Anderson 1978; Fei et al. (2009); Che et al. (2020)	Zhang et al. (2022)	Huang et al. (2023)

*Raorchestesgryllus* is still considered a member of *Raorchestes* in Frost (2023), although Poyarkov et al. (2021) suggested that it should be transferred to the genus *Kurixalus*. *Raorchesteshekouensis* sp. nov. can be distinguished from *K.gryllus* based on the following characters: no webbing between fingers (vs rudimentary webbing between fingers), rudimentary webbing between toes (vs little more than half webbed), heel with no pointed appendage (vs heel with small, pointed appendage), snout rounded (vs snout pointed with dermal tip), and series of tubercles along outer side of forearm and foot absent (vs present). *Raorchesteshekouensis* sp. nov. differs from *R.malipoensis* by inner metacarpal tubercle present, outer metacarpal tubercle indistinct (vs inner and outer metacarpal tubercle indistinct), heels meeting when limbs held at right angles to body (vs heels not meeting when limbs held at right angles to body); and relative toe lengths: I < II < III < V < IV (vs I < II < V < III < IV). *Raorchesteshekouensis* sp. nov. is distinguishable from *R.huanglianshan* by supernumerary tubercles absent (vs present) and lateral dermal fringes on all fingers and toes present (vs absent). *Raorchesteshekouensis* sp. nov. differs from *R.parvulus* by length of lower arm and hand slightly shorter than half of body size (vs longer than half of body size) and supernumerary tubercles absent (vs present on third finger). *Raorchesteshekouensis* sp. nov. differs from *R.menglaensis* by external single subgular vocal sac in adult male (vs internal single subgular vocal sac), tympanum distinct in male (vs indistinct), and lateral dermal fringes on all fingers and toes present (vs absent). *Raorchesteshekouensis* sp. nov. differs from *R.cangyuanensis* by tympanum distinct in male (vs indistinct) and relative toe lengths: I < II < III < V < IV (vs I < II < V < III < IV). *Raorchesteshekouensis* sp. nov. differs from *R.hillisi* by head wider than long (vs head longer than wide) and lateral dermal fringes on all fingers and toes present (vs fingers lacking lateral dermal fringes and toes with weak lateral dermal fringes, except outside of toe I and both sides of toe II). *Raorchesteshekouensis* sp. nov. differs from *R.dulongensis* by head wider than long (vs head longer than wide), snout rounded (vs pointed), relative toe lengths: I < II < III < V < IV (vs I < II < V < III < IV), and nuptial pad present (vs absent). *Raorchesteshekouensis* sp. nov. differs from *R.longchuanensis* by head wider than long (vs head length almost equal to width) and lateral dermal fringes on all fingers and toes (vs lateral dermal fringes only on fingers I and II and no lateral dermal fringes on toes). *Raorchesteshekouensis* sp. nov. differs from *R.andersoni* by tibiotarsal articulation reaching anterior of eye (vs tibiotarsal articulation reaching tip of snout), ventral surface of throat, chest, and belly opaque creamy white, with small black spots (vs chest and belly yellowish, with brown punctuations), and flank near crotch with distinct black region between two creamy white patches (vs irregular large black patch on groin, extending to half of side, with two yellow patches). *Raorchesteshekouensis* sp. nov. differs from *R.yadongensis* by lacking webbing between fingers (vs fingers with rudimentary webbing) and tibiotarsal articulation reaching anterior of eye when adpressed (vs tibiotarsal articulation reaching tip of snout when adpressed).

*Raorchesteshekouensis* sp. nov. differs from *R.rezakhani* by nuptial pad present (vs absent), dermal fringes present on fingers (vs absent), rudimentary webbing between toes (vs webbing moderate, formula: I2-2^+^II1¾-2^+^III1½-3IV2¾-2-V), and inner metacarpal and inner metatarsal tubercles present (vs absent). *Raorchesteshekouensis* sp. nov. differs from *R.annandalii* by snout rounded (vs pointed), supernumerary tubercles in toes absent (vs present), and inner metatarsal tubercle present (vs absent). *Raorchesteshekouensis* sp. nov. differs from *R.shillongensis* by inner metatarsal tubercles distinct, outer metatarsal tubercle absent (vs inner metatarsal tubercle indistinct, outer metatarsal tubercle present), and relative toe lengths: I < II < III < V < IV (vs I ≤ II < V ≤ III < IV). *Raorchesteshekouensis* sp. nov. differs from *R.sahai* by rudimentary webbing between toes (vs nearly half-webbed in toes) and mid-dorsal line absent (vs dark narrow line originating from interorbital region and extending posteriorly to hindmost part of body). *Raorchesteshekouensis* sp. nov. differs from *R.manipurensis* by rudimentary webbing between toes (vs almost 2/3 webbing in toes) and webbing between fingers absent (vs present).

### ﻿Key to *Raorchestes* species in China

**Table:** 

1	Fingers with rudimentary webbing	***R.yadongensis***
–	Fingers without webbing	**2**
2	Tympanum indistinct	**3**
–	Tympanum distinct	**4**
3	Fingers and toes with lateral dermal fringes	***R.cangyuanensis***
–	Fingers and toes lacking lateral dermal fringes	***R.menglaensis***
4	Internal single subgular vocal sac	***R.andersoni***
–	External single subgular vocal sac	**5**
5	Nuptial pad absent	***R.dulongensis***
–	Nuptial pad present	**6**
6	Toes with one-fourth webbing	***R.longchuanensis***
–	Toes not with one-fourth webbing	**7**
7	Fingers with lateral dermal fringe	**8**
–	Fingers lacking lateral dermal fringe	**9**
8	relative toe lengths: I < II < III < V < IV	***Raorchesteshekouensis* sp. nov.**
–	relative toe lengths: I < II < V < III < IV	***R.malipoensis***
9	Toes lacking lateral dermal fringe	***R.huanglianshan***
–	Toes with weak lateral dermal fringes, except outside of toe I and both sides of toe II	***R.hillisi***

## ﻿Discussion

**Figure 10.**
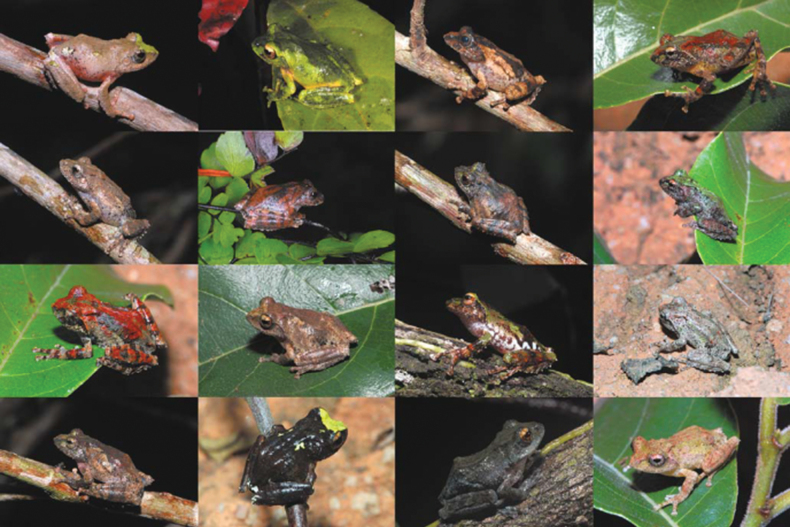
*Kurixalusgryllus* from Dak Lak Province (Chu Yang Sin National Park) and Lam Dong Province (Bidoup-Nui Ba National Park) in southern Vietnam (sourced from Orlov et al. 2012).

The small body size, morphological conservativeness, and remarkably similar characters in the *Raorchestes* genus have resulted in ambiguities in taxonomy and distribution (Jiang et al. 2020), necessitating the application of molecular identification (Orlov et al. 2012). Morphologically, the types and topotypes of *Kurixalusgryllus* are very similar to other members of the genus due to the series of tubercles along the outer side of the forearm and feet, small pointed appendage on the heel, and pointed snout with a dermal tip (Smith 1924; Orlov et al. 2012; Poyarkov et al. 2021), with wide variation in living color patterns of *K.gryllus* from the type locality shown to be very similar to that seen in *Kurixalusmotokawai* and *Kurixalusbanaensis* (Nguyen, 2015; see Fig. 10). Therefore, we agree with Poyarkov et al. (2021) that “*R.gryllus*” from the type locality should be reassigned to *Kurixalus*. We also consider that the samples of so-called “*R.gryllus*” from northern Vietnam used in the present study (ROM 38828 and ROM 30298) are not conspecific with *K.gryllus* from the type locality as they are phylogenetically nested within the genus *Raorchestes* (Fig. 2).

Our results showed that the *R.* UI ROM 38828 from northern Vietnam clustered with *Raorchesteshekouensis* sp. nov. with a short branch length, indicating that *R.* UI ROM 38828 belonged to the new species (Fig. 2), and recently Huang et al. (2023) revised the specimen ROM 30288 from northern Vietnam, which had been recorded as *R.gryllus*, to *R.malipoensis* so the taxonomic status of the *R.* UI specimen from northern Vietnam (ROM 30298) needs further confirmation. The genetic divergences between *R.malipoensis*, *R.* UI ROM 30298, and *Raorchesteshekouensis* sp. nov. were greater than the divergence between *R.hillisi* and *R.yadongensis* (Table 3), and species delimitations grouped them into three different candidate species (Fig. 3), indicating that the clade comprised of ROM 30298 likely represented an unnamed species, pending further morphological study. Of note, both the ROM 38828 and ROM 30288 specimens were collected from Pac Ban, Tuyen Quang, Vietnam, suggesting the coexistence of *R.malipoensis* and the new species *Raorchesteshekouensis* sp. nov. in that region, which means the records of *Raorchestes* from that region also need verification.

In this study, we used distance-based (ASAP) and tree-based (bPTP) delimitation methods, and the two different species delimitation methods give the same results. The ASAP analysis divides species based on pairwise genetic distance, but it can provide a score for each partitioning result for users to refer to and select partitioning results. The difference is that bPTP delimits species using non-hypermetric phylogenies, and estimates speciation events in terms of a number of substitutions; therefore, it only requires a standard phylogenetic tree as input. The combination of both methods confirms the species delimitation and helps overcome the constraints of each approach (Carstens et al. 2013).

With the description of the new species, there are now ten *Raorchestes* species known from China, all of which occur in Yunnan except for *R.yadongensis*, which is only known from southern Tibet, China (Zhang et al. 2022). Recently Garg et al. (2021) assigned the *Raorchestes* species into 16 species groups and the clade containing species from Southeast and East Asia (e.g., *R.parvulus*, *R.menglaensis*, *R.cangyuanensis*) was placed in the *R.parvulus* species group. Based on Garg et al. (2021) and our phylogenetic results, the new species also belongs to the *R.parvulus* group. The continuous discovery of new *Raorchestes* species from Yunnan in recent years (Wu et al. 2019, 2021; Jiang et al. 2020; Huang et al. 2023; this study) indicates that *Raorchestes* diversity is seriously underestimated in Yunnan. We expect that more *Raorchestes* species will be found from southern Yunnan given the unnamed lineage in adjacent northern Vietnam mentioned above, from Tam Dao, Vinh Phuc (ROM 30298). Therefore, further studies employing a wider range of *Raorchestes* samples across its distribution are necessary to clarify the species boundary in Yunnan.

Due to the placement of “*R.gryllus*” sensu stricto in *Kurixalus*, the number of recognized *Raorchestes* species known from Southeast Asia is decreased to five, including *R.parvulus*, *R.longchuanensis*, *R.menglaensis*, *R.malipoensis*, and *R.huanglianshan* based on recent studies (Poyarkov et al. 2021; Jiang et al. 2020; Wu et al. 2022; Huang et al. 2023); our results revealed the existence of an additional but unnamed lineage in northern Vietnam. Previous phylogenetic analyses have also revealed that nominal *R.parvulus*, which is widely reported across Indochina (Frost 2023), also contains multiple clades that do not form a monophyly (Chan et al. 2018; Wu et al. 2019; Yu et al. 2019; Jiang et al. 2020), indicating that multiple cryptic species may exist within the species. Therefore, *Raorchestes* species diversity in Southeast Asia may be highly underestimated.

## Supplementary Material

XML Treatment for Raorchestes hekouensis

## ﻿Additional information

### Author ORCIDs

Lingyun Du https://orcid.org/0000-0002-5761-4017

Shuo Liu https://orcid.org/0000-0001-7825-3006

Guohua Yu https://orcid.org/0000-0002-0220-6550

### Data availability

All of the data that support the findings of this study are available in the main text.
